# Surgery is more cost-effective than splinting for carpal tunnel syndrome in the Netherlands: results of an economic evaluation alongside a randomized controlled trial

**DOI:** 10.1186/1471-2474-7-86

**Published:** 2006-11-16

**Authors:** Ingeborg BC Korthals-de Bos, Annette AM Gerritsen, Maurits W van Tulder, Maureen PMH Rutten-van Mölken, Herman J Adèr, Henrica CW de Vet, Lex M Bouter

**Affiliations:** 1 Institute for Research in Extramural Medicine, VU University Medical Center, Amsterdam, the Netherlands; 2 Institute of Health Sciences, Faculty of Earth & Life Sciences, Vrije Universiteit, Amsterdam, the Netherlands; 3 Institute for Medical Technology Assessment, Erasmus Medical Center, Rotterdam, the Netherlands; 4 Department of Clinical Epidemiology and Biostatistics, VU University Medical Center, Amsterdam, the Netherlands

## Abstract

**Background:**

Carpal tunnel syndrome (CTS) is a common disorder, often treated with surgery or wrist splinting. The objective of this economic evaluation alongside a randomized trial was to evaluate the cost-effectiveness of splinting and surgery for patients with CTS.

**Methods:**

Patients at 13 neurological outpatient clinics with clinically and electrophysiologically confirmed idiopathic CTS were randomly allocated to splinting (n = 89) or surgery (n = 87). Clinical outcome measures included number of nights waking up due to symptoms, general improvement, severity of the main complaint, paraesthesia at night and during the day, and utility. The economic evaluation was performed from a societal perspective and involved all relevant costs.

**Results:**

There were no differences in costs. The mean total costs per patient were in the surgery group EURO 2,126 compared to EURO 2,111 in the splint group. After 12 months, the success rate in the surgery group (92%) was significantly higher than in the splint group (72%). The acceptability curve showed that at a relatively low ceiling ratio of EURO 2,500 per patient there is a 90% probability that surgery is cost-effective.

**Conclusion:**

In the Netherlands, surgery is more cost-effective compared with splinting, and recommended as the preferred method of treatment for patients with CTS.

## Background

Carpal tunnel syndrome (CTS), caused by compression of the median nerve at the wrist. In the Netherlands, the prevalence of electrophysiologically confirmed CTS in the adult general population was found to be 0.6% in men and 3.4% in women [1]. In Sweden, prevalence was 2.1% and 3.0%, respectively [2]. Patients with CTS are often treated with surgery or conservative methods of treatment (e.g. wrist splinting). A systematic review showed that open carpal tunnel release was the most suitable surgical technique [3]. One Cochrane review found that a hand brace and carpal bone mobilisation significantly improved symptoms and ultrasound treatment, oral steroid treatment and yoga significantly reduced pain [4]. Another Cochrane review demonstrated clinical improvement of symptoms of carpal tunnel syndrome at one month following local corticosteroid [5]. Two recent systematic reviews confirmed these findings [6,7]. There is still no consensus on whether surgery or conservative treatment is the best approach for patients with CTS [8-10].

Only two articles reported on the cost-effectiveness of surgical treatment options for CTS. Both studies compared endoscopic carpal tunnel release with open carpal tunnel release, and were based on decision-analytic simulation models [8,9]. One study concluded that endoscopic carpal tunnel release seemed to be a cost-effective procedure [8]. The other study concluded that the two techniques had similar total costs [9]. No cost-effectiveness analysis has yet been published in which surgery is compared with a conservative treatment option.

Therefore, we performed an economic evaluation alongside a randomized-controlled trial (RCT), to evaluate the cost-effectiveness and cost-utility of surgery compared with splinting for patients with CTS. An evaluation of the clinical outcomes of this trial has been reported elsewhere [10].

## Methods

The medical ethics committees of the 13 participating hospitals approved the study protocol of this multicenter RCT. A detailed description of the design and an evaluation of the clinical outcomes of this trial have been reported elsewhere [11,12].

### Study population

From October 1998 to April 2000, patients were recruited by neurologists in 13 participating hospitals. Patients were included if they: (1) had clinical and electrophysiologically confirmed CTS, (2) were 18 years of age or older, (3) and were able to complete written questionnaires (in Dutch). Patients were excluded from the trial if they: (1) were previously treated with splinting or surgery, (2) had a history of wrist trauma or surgery, (3) had a history suggesting underlying causes of CTS (e.g. diabetes mellitus, pregnancy), (4) had clinical signs or symptoms, or electrophysiological findings suggesting conditions that could mimic CTS or interfere with its validation (e.g. cervical radiculopathy, polyneuropathy), or (5) had severe thenar muscle atrophy.

### Treatment

After giving written informed consent and undergoing a baseline assessment, patients were randomly allocated to either splinting or surgery. Patients allocated to splinting were referred to a plaster-cast technician, an occupational therapist or a home-care store, depending on the usual procedures of the hospital at issue. They received either a prefabricated splint (trademark Tricodur, Beiersdorf) or a custom-made splint (made of soft-cast), that immobilized the wrist in neutral position. Patients were instructed to wear the splint during the night for at least 6 weeks and during the day only if they wished to. There were no restrictions for the patients in their work or normal daily activities. After 6 weeks the neurologist discussed with the patient whether any further treatment was necessary (continued splinting, other conservative treatment options or surgery).

For patients allocated to surgery, an appointment was made with a general surgeon, neurosurgeon, plastic surgeon or orthopaedic surgeon for outpatient standard open carpal tunnel release surgery (without epineurotomy or internal neurolysis, depending on the usual procedures of the hospital at issue). The patient was encouraged to use the hand as much as possible, and no absolute period off work was recommended.

### Outcome assessment

At baseline and 3, 6 and 12 months after randomization patients visited the hospital and completed written questionnaires. Clinical outcome measures included: (1) general improvement scored by the patients on a 6-point ordinal transition scale, ranging from 'completely recovered' to 'much worse'. A priori, success was defined as 'completely recovered' or 'much improved', (2) number of nights waking up due to the symptoms during the past week, (3) severity of the main complaint, and paraesthesia at night and during the day, scored by the patient on an 11-point numerical rating scale, ranging from 0 'no symptoms' to 10 'very severe symptoms', (4) quality of life measured by means of the EuroQol, and expressed as utility (0–1) [13].

### Economic evaluation

The aim of the economic analysis was to determine and to compare the total costs related to CTS for patients treated with splinting and for patients treated with surgery. Firstly, relevant categories of resource utilization were identified. Secondly, the volume of each category was measured, and these volumes were multiplied by the resource costs.

A societal perspective was the basis for the economic evaluation. Direct health care costs, direct non-health care costs and indirect costs due to CTS were used as economic indicators. Direct health care costs included the costs of the treatments (i.e. number of visits to medical specialist, operation, and wrist splint), additional visits to a health care provider (general practitioner, allied health professional, medical specialist or other health care professional), prescribed medication and professional home care. Direct non-health care costs included costs of over-the-counter medication, paid and unpaid help, visits to an alternative therapist, time spent visiting a health care provider and travel expenses. Indirect costs of loss of production, due to CTS-related absence from work, or hours of inactivity, were also included.

Data regarding the use of all health care resources were assessed by means of four cost diaries per patient covering together the entire follow-up period of 12 months [14]. These diaries were completed by the patients and returned to the research assistant at the hospital, who checked the cost-diaries with the patients.

The direct health care and direct non-health care costs (Table 1) were estimated according to the recently published Dutch guidelines for cost analysis in health care research [15,16]. In cases where these guidelines did not apply, the tariffs of the Dutch Central Organization for Health Care Charges were used to estimate costs [17]. The costs of medication were estimated on the basis of prices stated by the Royal Dutch Society for Pharmacy [18]. The time spent by a patient when visiting a practitioner and unpaid help were also included in the cost calculations, for which a shadow price of EURO 7.94 per hour was used [15].

Indirect costs of loss of production due to CTS, for both paid and unpaid labor, were calculated. In the main analysis for paid labor, these costs were calculated according to the Human Capital Approach, based on a mean income of the Dutch population according to age and gender of employees. For unpaid labor (e.g. voluntary work or household work), the indirect costs were estimated using a shadow price of EURO 7.94 per hour [15].

### Statistical analysis

The economic evaluation was carried out according to the intention-to-treat principle, i.e. the patients remained in the group they were randomly allocated to at baseline. As the percentage of missing cost data was only 9%, missing cost estimates were substituted by the mean of the measured estimates from the patient at issue in the previous period. To compare costs between groups, bootstrap confidence intervals were computed. The 95% confidence intervals were obtained by bias-corrected and accelerated (BCa) bootstrapping, choosing 2000 as the number of replications [19].

A cost-effectiveness analysis was performed, in which the primary clinical outcomes of the trial were expressed as mean improvement within each treatment group between baseline and 12 month's follow-up. A cost-utility analysis was also performed, in which the effects were expressed as utilities, based on the EuroQol. The cost-effectiveness and cost-utility ratios were calculated by dividing the difference between the mean costs by the difference between the mean improvement in the clinical outcomes. Cost-effectiveness ratio and cost-utility ratio were calculated with bootstrapping according to the bias-corrected percentile method [20]. The bootstrapped cost-effect pairs were graphically represented on a cost-effectiveness plane. Acceptability curves were calculated, which show the probability that a treatment is cost-effective at a specific ceiling ratio [21].

### Sensitivity analysis

Patients experienced difficulties in specifying the precise number hours of unpaid help. Because of this uncertainty, the influence of this cost-category on the total costs was evaluated. In the first sensitivity analysis neither the costs for unpaid help nor the costs for absenteeism from unpaid labor were included. In the second analysis, only costs for unpaid help were included. In the third analysis, the indirect costs for paid labor were calculated according to the Friction Cost Approach [22]. The basic idea of the FCA is that the amount of production loss (and/or the costs of maintaining production) due to sick leave, will depend on the time-span needed to restore the initial level of production and costs. Sick employees can be replaced after a necessary period of adaptation, the so-called friction period, which was estimated to be 122 days in the Netherlands [22].

## Results

**Figure 1:**
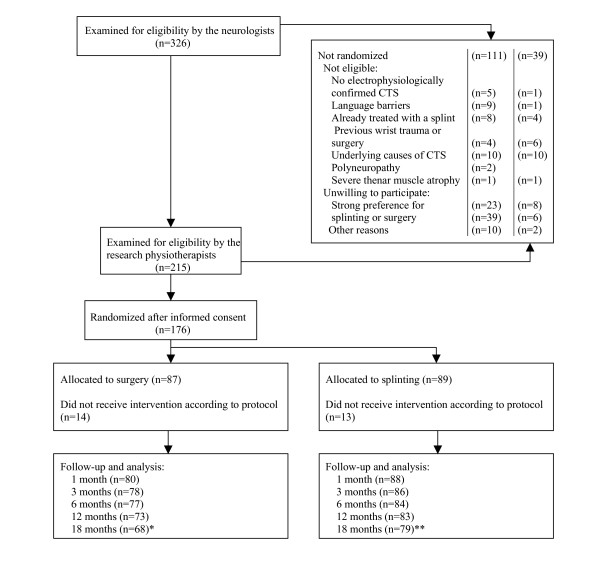
**Progress of patients through Carpal Tunnel Syndrome (CTS) trial**. * Reasons for withdrawal from the study (n = 19): refused to undergo surgery (n = 8); private matters (n = 2); lack of time (n = 1); died of cancer (n = 1); unable to attend the hospital at 12 months (n = 2); did not return the final questionnaire (n = 5). ** Reasons for withdrawal from the study (n = 10): lack of time (n = 3); private matters (n = 1); not satisfied with treatment result (n = 1); unable to attend the hospital at 12 months (n = 1); did not return the final questionnaire (n = 4).

A total of 176 patients was included in the trial, and they were randomized to splinting (n = 89) and surgery (n = 87). Overall, 83 (93%) in the splint group and 73 (84%) in the surgery group completed the follow-up measurement 12 months after randomization. The main reason for withdrawal in the splint group was 'lack of time', and in the surgery group the main reason was 'unwillingness to undergo surgery' (Figure 1). Every patient had to fill in four cost diaries, covering together the entire 12 months follow-up period, resulting in a total of 704 cost diaries. Only 61 cost diaries (9%) were not returned. In total from 79 patients in the surgery group and from 88 patients in the splint group were cost data available.

### Clinical effects

The success rates (based on the outcome measure general improvement) and improvement in the other clinical outcome measures at 12 months are presented in Table 2. The success rate in the surgery group (92%) was significantly higher than in the splint group (72%). Surgery was also significantly superior to splinting with regard to severity of the main complaint and paraesthesia during the day. There were no statistically significant differences with regard to number of nights waking up due to symptoms and paraesthesia at night. The utility in the surgery group after 12 months was 0.85 (SD: 0.12), compared to 0.81 (SD: 0.16) in the splint group, not statistically significant difference.

### Utilization of health care resources

The utilization of health care resources and work absenteeism during the follow-up period of 12 months is presented in Table 3. Visits to health care providers were mainly restricted to medical specialist care. The patients in the splint group visited medical specialists more often than the patients in the surgery group, because a number of patients underwent surgery after their initial treatment with a wrist splint. Patients in both groups seldom visited a general practitioner or an allied health professional. Only one patient in the surgery group and two patients in the splint group visited an alternative therapist. Twelve patients in the splint group used medication on prescription, compared to sixteen patients in the surgery group. The number of patients using over-the-counter medication was higher in the surgery group (n = 35; 44%) than in the splint group (n = 20; 22%). In the surgery group 73 of the 87 patients underwent surgery, while fourteen patients refused to undergo the operation. All patients in the splint group received a splint at the beginning of the trial. After one year, 33 patients (39%) in the splint group had also undergone surgery.

The number of hours that patients received unpaid help was high in both groups. In the surgery group 42 patients (53%) received unpaid help, compared to 26 patients (30%) in the splint group.

At baseline, 50 patients (57%) in the surgery group had a paid job, compared to 53 patients (60%) in the splint group. During the trial, 34 patients in the surgery group had been absent from paid labor (mean of 12.1 days). One patient had a very long period of work absenteeism of 248 days. Twenty patients in the splint group had been absent from work (mean of 11.8 days). In the splint group, four patients had long periods of work absenteeism, varying from 120 to 260 days. If those five patients with long periods of work absenteeism were excluded from the analysis, the mean number of days of absenteeism of those who had been absent from paid labor in the surgery group decreased from 12.1 to 9.2 days and in the splint group from 11.8 to 3.1 days.

The findings were similar for absenteeism from unpaid labor: 46 patients in the surgery group, compared to 36 patients in the splint group could not perform their normal daily activities for approximately 50 hours during the 12 months of the trial.

### Costs

Table 4 shows the mean costs per treatment group and the differences in mean costs between the groups during the follow-up period of 12 months. The mean direct health care costs were statistically significantly lower for surgery. The main contributor to the direct non-health care costs was the cost of help from family and friends. The total direct costs in the surgery group were lower, compared to the splint group. The indirect costs in the surgery group were EURO 1,544 compared to EURO 1,427 in the splint group. The total costs were EURO 2,126 in the surgery group and EURO 2,111 in the splint group.

### Cost-effectiveness and cost-utility analyses

**Figure 2:**
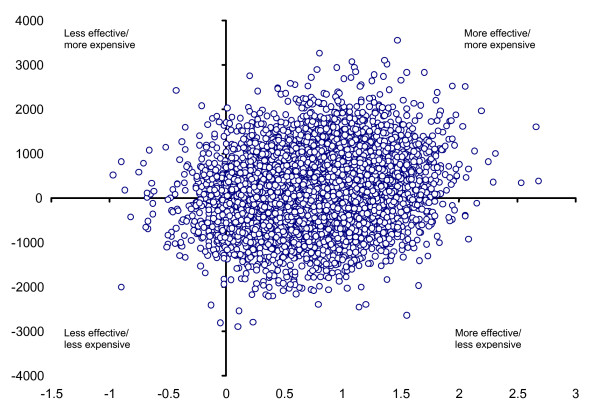
Cost-effectiveness plane comparing surgery and splinting for number of nights awake due to symptoms.

**Figure 3:**
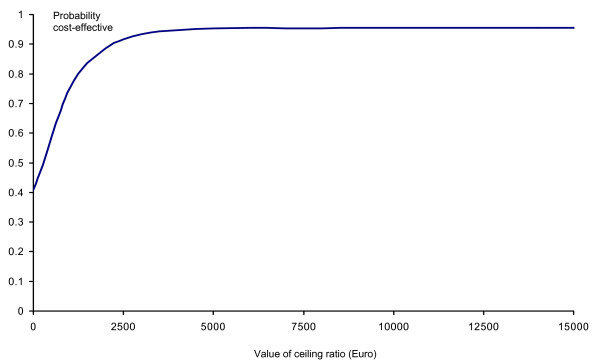
**Acceptability curve for surgery vs. splinting for number of nights awake due to symptoms**. At a ceiling ratio of EURO 2500 per patient there is a 90% probability that surgery is cost-effective.

In Table 5 the cost effectiveness and cost utility ratios are presented. The cost-effectiveness ratio for surgery compared to splinting for 'number of nights waking up due to symptoms' was EURO -40, meaning that surgery was EURO 40 cheaper in achieving one time less waking up at night than splinting. Figure 2 shows the cost-effectiveness plane for this outcome measure. 56% of the incremental cost/effect pairs are located in the north-east quadrant and 39% are located in the south-east quadrant. The other outcome measures showed similar results. Surgery was statistically significantly more effective than splinting. Because the total costs of both interventions were similar, the cost-effectiveness ratios did not provide additional information. In Figure 3 the acceptability curve for number of nights waking up due to the symptoms is presented for surgery compared to splinting. This curve shows that at a relatively low ceiling ratio of EURO 2,500 per patient there is a 90% probability that surgery is cost-effective.

### Sensitivity analysis

In the sensitivity analyses the cost-categories included in the direct non-health care costs and the indirect costs were varied. Many patients reported unpaid help as well as absenteeism from unpaid work. In the first sensitivity analysis the costs of help from family and friends and the costs of absenteeism from unpaid labor were excluded, and this obviously resulted in lower direct non-health care, indirect and total costs. However, this did not influence the results. The other sensitivity analyses also showed no substantial changes in the results (data not shown). The sensitivity analysis using the Friction Cost Approach, in which the maximum friction period of 122 days was used for the five patients with long periods of work absenteeism, did not change the results.

## Discussion

In this trial, the cost-effectiveness and cost-utility of two commonly applied methods of treatment for CTS was evaluated. The results of the intention-to-treat analyses showed that after 3 and 6 months surgery was clearly more effective than splinting on all outcome measures [11]. After 12 months, the success rate in the surgery group was 92% and in the splint group 72%.

The mean direct health care costs in the surgery group were a little bit lower than in the splint group. This was due to the fact that a substantial number of patients in the splint group received surgery after their treatment with a wrist splint. Furthermore, in the Netherlands the cost of open carpal tunnel release surgery is even lower than the cost of a wrist splint. These two facts contributed to lower direct health care costs in the surgery group. Some patients in the splint group received a custom-made splint, and others a prefabricated splint. The cost of a visit to the person who makes the custom-made splint or provides the prefabricated splint was calculated to be the same as a visit to a medical specialist. However, in the Netherlands patients can also obtain a standard splint in a home-care store where no extra charges are made.

In this trial, different types of surgeons operated on the patients. In the Netherlands the standard tariff for an intervention is increased with a percentage that varies according to the specialism of the surgeon (i.e. general surgeon 24.5%, neurosurgeon 43.5%). Only the standard tariff for a general surgeon was used in this study, instead of different tariffs for different types of surgeons. The direct non-health care costs and the total direct costs were lower in the surgery group than in the splint group, but these differences were not statistically significant.

The mean total costs after 12 months were EURO 2,126 (SD 4,618) in the surgery group and EURO 2,111 (SD 5,568) in the splint group. Consequently, the outcome measures are decisive which treatment option should be given to patients with CTS. This randomized controlled trial with an economic evaluation showed that, compared to splinting, surgery had better clinical effects and there were no difference in the costs. Therefore, on the basis of the results of this study, surgery is clearly the superior method of treatment for patients with CTS. This is not in line with the recommendation of The American Academy of Neurology that advises treatment of CTS with non-invasive options (e.g. splinting) initially, and OCTR only if non-invasive treatment proves to be ineffective [23].

A limitation of this study is that the results may be limited in how they can be applied to other countries in which the costs of surgery would be much higher. As surgery is much more expensive in other countries than in the Netherlands, results of this study may not be directly extrapolated. It is important to realize that the costs used in an economic evaluation are the real costs of the intervention and not the price that is paid by either the (public or private) health insurers and/or the patients. Not the prices charged for this intervention, but the real cost price should be included as the costs of the intervention in the economic evaluation. The real cost price is based on personnel, material and overhead costs. This intervention is a simple outpatient intervention, the surgeon and nurse don't spend more than 10–15 minutes to perform the intervention, and material costs are low. Assuming a salary of Euro 200 per hour for the surgeon and 50 for the nurse, the total personnel costs would be Euro 250/4 (assuming 15 minutes per intervention), which equals Euro 62.50. So, the tariff of Euro 69.50 we used seems a good proxy for the real cost price. The prices charged for this intervention in other countries are much higher than the actual cost price, which indicates that profits are (very) high. Economic evaluations in other health care systems (countries) are recommended, and these economic evaluations should also use the real cost price and not charges.

## Conclusion

In the Netherlands, surgery is more cost-effective compared with splinting, and recommended as the preferred method of treatment for patients with CTS.

## Competing interests

The author(s) declare that they have no competing interests.

## Authors' contributions

MvT, MR, HdV and LB were responsible for conception and design of the study. IK, AG, MvT, MR and HA were involved in analysis and interpretation of data. IK, AG and MvT were responsible for drafting the article and revising it critically. All authors read and approved the final manuscript.

## Pre-publication history

The pre-publication history for this paper can be accessed here:



## Figures and Tables

**Table 1:** Prices used in the economic evaluation

	EURO
**Direct health care costs**	
Outpatient appointment	40.85
Hospitalisation (per day)	235.95
Operation carpal tunnel syndrome	69.50
Splint	72.10
	
General practitioner (visit of max. 20 min.)	16.60
Manual therapist (visit of max. 45 min.)	25.90
Physical therapist (visit of max. 30 min.)	18.15
Cesar exercise therapist (per visit)	17.70
	
Professional home care (per hour)	22.70
	
**Direct non-health care costs**	
Unpaid help (per hour)	7.94
Time spent visiting a health care provider (per hour)	7.94
	
**Indirect costs**	
Absenteeism paid labour (per day) *	-
Absenteeism unpaid labour (per hour)	7.94

Euro 1 = US $0.90; * Costs for paid labour were calculated on the basis of a mean income of the Dutch population according to age and sex.^[14,15]^

**Table 2:** Primary outcome measures for the treatment groups after 12 months

Primary outcome measures	Surgery (n = 73)	Splint (n = 83)	Difference*
Success rate (%)**	92% (67/73)	72% (60/83)	20% (8; 31)
# nights waking up due to complaints (0–7)**	3.6 (2.9)	2.9 (3.0)	0.7 (-0.2; 1.7)
Severity of the main complaint (0–10)**	6.4 (2.7)	5.1 (3.1)	1.3 (0.4; 2.2)
Paraesthesia during the day (0–10)**	5.5 (2.9)	4.0 (3.4)	1.5 (0.5; 2.5)
Paraesthesia at night (0–10)**	5.2 (3.6)	4.5 (3.4)	0.7 (-0.4; 1.8)
Utility (EuroQol; 0–1)***	0.85 (0.12)	0.81 (0.16)	0.04 (-0.004; 0.08)

*Presented are the difference in success rate and the differences in the mean improvements from baseline (95% confidence interval); **Presented are the success rate and the mean (standard deviation) improvements from baseline; ***Utility is scored for one year.

**Table 3:** Utilisation of health care resources and work absenteeism per treatment group during 12 months follow-up

Type of utilisation [Unit of measurement]	Surgery* (n = 79)	Splint* (n = 88)
Medical specialist care [no. of outpatient visits]**	2.5 (1.4)	3.3 (1.8)
General practice [no. of visits]	0.7 (1.5)	0.3 (0.7)
Allied health professions [no. of treatment sessions]***	0.7 (4.6)	0.8 (3.4)
Professional home care [no. of hours]	0.3 (2.3)	0.5 (3.3)
Unpaid help [no. of hours]	40 (151)	39 (110)
Absenteeism paid labour [no. of days]	12.1 (31)	11.8 (42.8)
Absenteeism unpaid labour [no. of hours]	50 (141)	52 (143)

*Presented are the mean (standard deviation); ** Including the number of visits for the randomly allocated intervention; *** Allied health professions are physiotherapy, manual therapy, exercise therapy.

**Table 4:** Mean total costs (in EURO) during 12 months follow-up (main analysis)*

Costs	Surgery (n = 79)	Splint (n = 88)	Difference **
Direct health care costs	216 (161)	273 (163)	-57 (-103;-10)
Direct non-health care costs	366 (1,213)	412 (1,124)	-46 (-379;325)
Total direct costs	582 (1,256)	684 (1,198)	-103 (-472;316)
Indirect costs	1,544 (3,508)	1,427 (4,514)	118 (-1,034;1,448)
Total costs	2,126 (4,618)	2,111 (5,568)	15 (-1,458;1,913)

EURO 1 = US $0.90; * Presented are the mean (standard deviation) costs; ** Presented are the differences in mean costs between the treatment groups (95% confidence interval obtained by bias corrected and accelerated bootstrapping).

**Table 5:** Cost effectiveness and cost utility ratios (in EURO) (main analysis)*

Costs	Surgery (n = 73)**		Splint (n = 82)**		Ratio
			
	Costs	Effects	Costs	Effects	
# Nights waking up due to complaints (0–7)	2,215	3.6	2,244	2.9	-40
Severity of the main complaint (0–10)	2,215	6.4	2,244	5.1	-21
Paraesthesia during the day (0–10)	2,215	5.5	2,244	4.0	-19
Paraesthesia at night (0–10)	2,215	5.2	2,244	4.5	-40
Utility (EuroQol; 0–1)***	2,126	0.85	2,111	0.81	353

EURO 1 = US $0.90; *Presented are the mean (standard deviation) improvements from baseline; ** Patients with missing data on either outcome measures or costs are excluded; *** Number of patients for utility in surgery (n = 79) and splint (n = 88).
